# Corrosion, Fatigue and Corrosion Protection of Metals and Their Alloys in Various Environments

**DOI:** 10.3390/ma19091770

**Published:** 2026-04-27

**Authors:** Florina Branzoi

**Affiliations:** Institute of Physical Chemistry “Ilie Murgulescu”, 202 Splaiul Independenţei, 060021 Bucharest, Romania; fbrinzoi@chimfiz.icf.ro

The continued reliance on metallic materials across industrial, energy, transportation, biomedical, and infrastructural sectors underscores the persistent importance of understanding and mitigating degradation phenomena that compromise structural integrity and operational reliability. Corrosion, wear, and fatigue remain among the most pervasive and costly challenges associated with metallic systems, particularly as modern applications demand higher performance under increasingly aggressive environmental and mechanical conditions. The global shift toward lightweight structures, high-efficiency energy systems, and environmentally responsible technologies has intensified the need for materials capable of withstanding complex combinations of chemical, electrochemical, mechanical, and biological stressors. As industries evolve, the environments in which metallic materials operate become more severe, more variable, and more difficult to predict, requiring a deeper understanding of the mechanisms that govern degradation and the development of advanced strategies for protection [1,2,3,4,5,6,7,8,9,10,11,12,13,14,15,16,17,18,19,20].

In this context, the scientific community continues to expand the boundaries of corrosion science and surface engineering, exploring not only the fundamental mechanisms that drive material deterioration but also the innovative processing routes, protective coatings, inhibitors, and surface treatments that can extend service life. The interplay between microstructure, surface condition, environmental chemistry, and mechanical loading has emerged as a central theme in contemporary materials research. It is increasingly evident that no single factor governs the durability of metallic systems; rather, performance arises from the convergence of multiple, interdependent variables that must be understood holistically [21,22,23,24,25,26,27,28,29,30,31,32,33,34,35,36,37,38,39,40]. This Special Issue, *Corrosion, Fatigue and Corrosion Protection of Metals and Their Alloys in Various Environments*, brings together ten contributions that collectively reflect the breadth and depth of current research in this field. The articles span a wide spectrum of materials—from aluminum and titanium alloys to carbon steels, stainless steels, and nickel–titanium systems—and address environments ranging from acidic and alkaline media to CO_2_-rich solutions, microbial ecosystems, and hydrodynamic cavitation conditions.

The editorial that follows synthesizes the key findings of each contribution, situating them within the broader landscape of corrosion and materials engineering. The aim is not only to highlight the scientific advances presented in this Special Issue but also to articulate the conceptual threads that connect them: the centrality of microstructural control, the importance of surface phenomena, the role of environmental chemistry, the emergence of biological influences, and the promise of innovative protective technologies. Together, these studies offer a comprehensive perspective on the challenges and opportunities associated with designing metallic materials for long-term service in diverse and demanding environments.

The issue opens with the investigation of heat-treated aluminum alloys, where the balance between mechanical performance and corrosion resistance is often delicate. In their study on the A356 Al–Si casting alloy, Jang et al. [41] demonstrate how solution treatment and aging refine the morphology of Si particles and promote the precipitation of Mg_2_Si, resulting in a substantial increase in hardness. Yet this microstructural optimization introduces a critical trade-off: the same Mg_2_Si precipitates that enhance mechanical strength also reduce corrosion resistance by depleting magnesium from the aluminum matrix. Their work illustrates the need for carefully balanced heat-treatment strategies that consider both mechanical and electrochemical performance, a theme that resonates throughout the Special Issue.

A complementary perspective on aluminum alloys is provided by Rong et al. [42], who examine AlSi10Mg materials fabricated through Spark Plasma Sintering. Their results show that the sintering temperature governs grain refinement, eutectic silicon dispersion, and ultimately the alloy’s wear and corrosion behavior. Optimal wear resistance is achieved at 525 °C due to the uniform precipitation of fine Si phases, while the best electrochemical performance occurs at 500 °C, where the microstructure minimizes galvanic interactions. This study reinforces the idea that processing parameters are powerful tools for tailoring microstructure-sensitive degradation mechanisms and highlights the importance of integrating mechanical and electrochemical considerations in alloy design.

The importance of surface condition becomes even more evident in the work of Dobri et al. [43], who investigate multi-component titanium alloys containing tantalum. Their long-term immersion tests in phosphate-buffered saline reveal that alloys with 10–20% Ta exhibit excellent passivation behavior, but only when the surface is smooth. Rough surfaces, even with tantalum additions, show diminished performance, indicating that surface topography can amplify electrochemical heterogeneities and disrupt passive film stability. This synergy between alloy chemistry and surface morphology is particularly relevant for biomedical applications, where long-term implant performance depends on the integrity of passive films in physiological environments.

A shift toward mechanistic understanding is offered by Hur et al. [44], who elucidate a previously unrecognized pathway for nitrite-based inhibition of carbon steel corrosion in alkaline environments. Their work demonstrates that nitrites undergo electrochemical reduction to ammonium ions, which in turn promotes the formation of a thin, protective Fe_3_O_4_ film. This mechanism explains the dramatic reduction in corrosion rate observed in the presence of nitrites and challenges traditional assumptions about their role as simple oxidizing agents. By identifying the chemical species involved in film formation, this study refines our understanding of nitrite-based inhibitors and offers valuable insights for industrial systems that rely on alkaline corrosion control.

Organic inhibitors are explored in the contribution by Brânzoi et al. [45], who examine the protective action of Brij-type nonionic surfactants on OLC 45 carbon steel in sulfuric acid. Through electrochemical testing and surface characterization, they show that these surfactants adsorb spontaneously onto the steel surface, forming mixed-type inhibitive films that significantly reduce both anodic and cathodic reactions. The adsorption follows Langmuir behavior and involves both physisorption and chemisorption, with inhibition efficiencies reaching up to 96%. Their findings highlight the potential of molecularly engineered organic inhibitors as environmentally compatible alternatives for corrosion protection in acidic environments.

The complexity of localized corrosion is addressed by Hu et al. [46], who investigate crevice corrosion in N80 carbon steel exposed to CO_2_-saturated NaCl-HAc solutions. Contrary to classical expectations, the crevice environment becomes alkaline rather than acidic, leading to galvanic interactions between the inner and outer surfaces and resulting in severe attack near the crevice mouth. The severity of corrosion depends on crevice size, with smaller crevices (100–300 µm) exhibiting more aggressive behavior than larger ones. These findings challenge conventional models of crevice chemistry and emphasize the complex interplay between geometry, mass transport, and electrochemical gradients in localized corrosion processes.

Biological influences on corrosion are brought to the forefront by Sun et al. [47], who examine the behavior of L245 pipeline steel in the presence of sulfate-reducing bacteria. Their study shows that biofilm development initially suppresses uniform corrosion by increasing charge-transfer resistance, yet simultaneously promotes severe pitting beneath the film as bacteria shift to electron uptake directly from Fe^0^. This dual behavior, i.e., protection against general corrosion but acceleration of localized attack, captures the paradoxical nature of microbially influenced corrosion and underscores the need for integrated bio-electrochemical approaches in pipeline integrity management.

Mechanical degradation under extreme hydrodynamic conditions is explored by Cosma et al. [48], who investigate the cavitation erosion behavior of GX40CrNiSi25-20 stainless steel subjected to laser surface remelting. The remelted layer, characterized by fine grains and enhanced hardness, increases resistance to cavitation erosion by nearly fivefold compared to the untreated material. Their findings demonstrate how targeted surface engineering can dramatically improve performance in environments dominated by cavitation and highlight the importance of microstructural refinement for resisting mechanically driven degradation.

A different form of mechanical degradation is examined by Martins et al. [49], who focus on the cyclic fatigue strength of nickel–titanium (NiTi) endodontic instruments. In the narrow and curved environment of a root canal, these tools are subjected to intense mechanical stresses that may lead to sudden fracture. By analyzing five different rotary systems, the authors established a clear correlation between heat-treatment processes and fatigue life. Their results demonstrate that heat-treated NiTi alloys, which possess specific phase transformation temperatures, offer far greater durability than traditional versions. This work bridges the gap between metallurgical phase theory and clinical safety, ensuring that surgical instruments can withstand the rigorous demands of modern dentistry.

The Special Issue concludes with a comprehensive review by Protsenko [50], who examines corrosion-resistant coatings electrodeposited from deep eutectic solvent (DES) electrolytes. DES-based systems offer environmental, technological, and economic advantages over traditional aqueous or organic baths, enabling precise control over coating composition, morphology, and microstructure. The review highlights the potential of DESs for producing nanocrystalline and composite coatings with superior corrosion and wear resistance, while also identifying challenges related to scalability, long-term performance, and process standardization. This forward-looking perspective positions DES-assisted electrodeposition as a promising frontier in sustainable surface engineering.

Taken together, the contributions in this Special Issue illustrate the multifaceted nature of corrosion and degradation phenomena and the diverse strategies available to mitigate them. They demonstrate that the performance of metallic materials is governed not by isolated factors but by the interplay of microstructure, surface condition, environmental chemistry, mechanical loading, and biological activity. The studies presented here underscore the importance of interdisciplinary approaches in advancing corrosion science, integrating insights from metallurgy, electrochemistry, surface engineering, microbiology, and mechanical testing. As industries continue to demand materials that are lighter, stronger, more durable, and more environmentally compatible, the need for advanced protective strategies becomes increasingly pressing. The research compiled in this Special Issue provides a valuable foundation for future innovation, offering both mechanistic understanding and practical pathways for improving the longevity of metallic systems. Continued progress will depend on the development of integrated models that capture the complexity of real-world environments, the refinement of advanced processing and surface-engineering techniques, and the exploration of sustainable and scalable protective technologies.

As guest editor, I would like to express my sincere appreciation to all contributing authors for the depth, rigor, and originality of their work. Their studies collectively demonstrate the vitality of corrosion science as a field that continuously adapts to new industrial, environmental, and societal challenges. The diversity of approaches represented here, from microstructural engineering and inhibitor chemistry to biological interactions, cavitation resistance, and emerging electrochemical technologies, highlights the dynamic evolution of the discipline and its capacity to address increasingly complex degradation scenarios. It is my hope that the insights gathered in this Special Issue will stimulate further research, encourage cross-disciplinary collaboration, and support the development of materials and protective strategies capable of meeting the demanding conditions of modern technological systems.
